# Wealth and education-related inequalities in the utilisation of reproductive, maternal, newborn, and child health interventions within scheduled tribes in India: an analysis of Odisha and Jharkhand

**DOI:** 10.1186/s12889-024-18857-4

**Published:** 2024-06-17

**Authors:** Rekha S., Varshini Neethi Mohan, Girija Vaidyanathan, Umakant Dash, V. R. Muraleedharan

**Affiliations:** 1 Department of Humanities and Social Sciences (DoHSS), Indian Institute of Technology (IIT), Madras, India; 2 Institute of Rural Management Anand, Anand, Gujarat India

**Keywords:** Reproductive maternal newborn and child health, RMNCH, Scheduled tribes, ST, India, NFHS-5, India, Inequality, Composite coverage index, CCI, Co-coverage indicator

## Abstract

**Background:**

The utilisation of Reproductive, Maternal, Newborn and Child Health (RMNCH) services remains lower among the Scheduled Tribes (ST) in India than among the rest of the country’s population. The tribal population’s poorest and least-educated households are further denied access to RMNCH care due to the intersection of their social status, wealth, and education levels. The study analyses the wealth- and education-related inequalities in the utilisation of RMNCH services within the ST population in Odisha and Jharkhand.

**Methodology:**

We have constructed two summary measures, namely, the Co-coverage indicator and a modified Composite Coverage Index (CC), to determine wealth- and education-related inequalities in the utilisation of RMNCH indicators within the ST population in Odisha and Jharkhand. The absolute and relative inequalities with respect to wealth and education within the ST population are estimated by employing the Slope Index of Inequality (SII) and the Relative Index of Inequality (RII).

**Results:**

The results of the study highlight that access to RMNCH services is easier for women who are better educated and belong to wealthier households. The SII and RII values in the co-coverage indicator and modified CCI exhibit an increase in wealth-related inequalities in Odisha between NFHS-4 (2015-16) and NFHS-5 (2019-21) whereas in Jharkhand, the wealth- and education-related absolute and relative inequalities present a reduction between 2016 and 2021. Among the indicators, utilisation of vaccination was high, while the uptake of Antenatal Care Centre Visits and Vitamin A supplementation should be improved.

**Interpretation:**

The study results underscore the urgent need of targeted policies and interventions to address the inequalities in accessing RMNCH services among ST communities. A multi-dimensional approach that considers the socioeconomic, cultural and geographical factors affecting healthcare should be adopted while formulating health policies to reduce inequalities in access to healthcare.

Evidence before this study We conducted a literature search on trends regarding wealth-related inequalities in RMNCH care services utilization among tribal populations in India or other states using PubMed and Google Scholar. The terms used for the search include ‘wealth’ OR ‘wealth-related’ OR ‘wealth-based’ AND ‘inequalities’ AND ‘tribal population’ OR ‘Scheduled Tribes (ST) population’ OR ‘tribal women’ OR ‘Scheduled Tribes Women’ AND ‘India’. We have found studies that have determined trends in inequalities in RMNCH care services utilization among general population in India and different states in India. However, we did not identify any literature on trends in wealth-related inequalities RMNCH care utilization within ST communities in India.

Added value of this study Our study delves into the temporal dynamics and disparities in the utilization of RMNCH care services among Scheduled Tribes (ST) in Odisha and Jharkhand, utilizing unit-level data from two rounds of the National Family Health Survey (NFHS-4 (2015-16) and NFHS-5 (2019-21)). Employing the Slope Index of Inequality (SII) and Relative Index of Inequality (RII), we assess absolute and relative inequalities, respectively. To comprehensively analyze these disparities, we employ two summary indices: the coverage indicator, which evaluates the co-coverage rates of mothers and children by six or more out of eight RMNCH interventions, and the composite coverage index (CCI), modified for our study’s purposes, which offers a weighted average of coverage across the same set of interventions.

Implications of all available evidence The study results show a pro-rich pattern in the utilization of RMNCH care services in both Odisha and Jharkhand. There was an increase in both absolute and relative inequalities with respect to both wealth and education in Odisha between 2016 and 2021, while both wealth- and education-based absolute and relative inequalities decreased in Jharkhand between the two rounds of the NFHS. The results of this study provide evidence for policymakers to further focus on wealth- and education-based inequalities within these groups and devise suitable policy interventions to address gaps, specifically in the number of ANC visits.

## Background

Although maternal and child death rates have improved significantly worldwide since 1990 (49% and 45%, respectively), they remain disproportionately high in Low-and Middle-income countries. Around 300,000 mothers and 6.6 million children die annually from preventable causes, primarily belonging to poor countries [1–4]. Over the past three decades, the Maternal Mortality Rate (MMR) and Under-5 Mortality Rate (U5MR) in India have significantly declined. Evidence from the Sample Registration System (SRS) shows that the MMR decreased from 556 in 1990 to 113 in 2016-18 and UMR from 113 in 1990 to 36 in 2018 [5]. However, this reduction is not evenly distributed, and national and subnational disparities exist in the progress achieved [6].

In most societies, there is a stepwise or linear decrease in the population’s health with a reduction in their social position; this is termed the ‘social gradient in health’ [7]. It is regarded as a ‘shortfall’ in health and indicates the number of lives that could have been saved if everyone in the society had the same level of health [8, 9]. There exists a high level of pregnancy-related mortality and an underutilisation of essential maternal health services among marginalised communities across the world [10–12].

In India, communities classified as scheduled tribes (ST) are one such marginalised group with poorer health outcomes than the rest of the country’s population. ST communities are recognised under Article 366/342 of the Constitution of India, categorised based on primitive traits, distinctive culture, geographical isolation, shyness of contact with the community at large, and backwardness [13]. The ST population in India constitutes 8.6% of the country, which translates to 104 million tribal people [14]. Although numerous policies and programmes have been undertaken and carried out for their social and economic upliftment in post-independence India, all development indicators reveal that they are at the bottom of the social gradient in health [15].

There exist marked inequalities in access to RMNCH care depending on wealth and levels of education [16]. While several interventions aim to improve access to healthcare, specifically for people belonging to Scheduled Tribes, there exist considerable inequalities within this population as well. The existing literature has highlighted that the poor economic condition of women and education as the two most significant predictor variables for lower utilization of maternal health care services in India [17–23]. Intersectionality between social status and wealth, and education levels could lead to the poorest and the least educated of the tribal population being the most severely deprived of RMNCH care [15]. Based on this backdrop, we aim to determine the magnitude of wealth- and education-related inequalities in the utilization of RMNCH care services within the ST population.

Our study analysis within-group inequalities among ST communities in RMNCH coverage in the states of Odisha and Jharkhand. The study employs a novel approach using a secondary data source to estimate disparities or inequities prevalent among ST communities regarding wealth and education. To thoroughly examine these differences, we utilize two summary metrics, which provide a comprehensive analysis of RMNCH coverage disparities among ST communities, allowing for a nuanced understanding of access to these interventions. First, the coverage indicator, which assesses the extent to which mothers and children are covered by six or more out of eight RMNCH interventions, and second, the modified composite coverage index (CCI) tailored for our study, provides a weighted average of coverage across the same interventions.

We selected Odisha and Jharkhand because of their substantial representation of Scheduled Tribes within their populations. Among women who gave birth in the 5 years preceding NFHS-5, 24.65% in Odisha and 26.86% in Jharkhand belonged to Scheduled Tribes. Our analysis used data from the last two rounds of the National Family Health Survey (NFHS-4; 2015-16 and NFHS-5; 2019-21). Research on RMNCH discrepancies in Odisha and Jharkhand may yield insights that are relevant to other areas with comparable healthcare difficulties and demographic makeup. Consequently, the study’s geographic focus not only helps address healthcare inequities at the local level but also has the potential to inspire more comprehensive public health programmes that aim to improve RMNCH outcomes for marginalised people in various geographic settings.

The factors that lead to poor health outcomes and mortality among pregnant women are closely linked to those affecting their babies. For example, complications during labour can lead to maternal, foetal or newborn deaths and long-term disabilities in the child [24]. Access to quality antenatal, delivery and postnatal care, as well as a skilled workforce, appropriate supplies, effective referral systems, solid infrastructure, high levels of education in women, and other social determinants, have a positive impact on reducing and preventing stillbirths and maternal and newborn deaths [25]. We use summary measures of specific indicators to evaluate access to RMNCH services that might have an impact on mortality.

This paper is structured as follows: in Section. 2, we present the methodology of the study. Subsequently, in Section. 2.1, we present a comparison of the utilisation of RMNCH indicators using the co-coverage indicator and modified composite coverage index (modified CCI) between ST and non-ST populations in Odisha and Jharkhand. Section 2.2 classifies the ST population into wealth and education categories and presents the summary measures for both states for the two rounds of NFHS. Section 2.3 analyses the absolute and relative inequalities by wealth and education in co-coverage indicator and modified CCI among ST populations in Odisha and Jharkhand for the two rounds of NFHS. Section 2.4 breaks down the CCI and the co-coverage indicator into their components and employs equiplots to represent inequalities visually. A heatmap for the indicators is also presented here. We conclude with a discussion of the implications of the study results in Section. 3. In Section. 4, the conclusion of the paper is presented.

## Methodology

### Data source

The study uses the latest two rounds of the National Family Health Survey (NFHS-4 (2015-16) and NFHS-5 (2019-21)) for analysis. The National Family Health Survey (NFHS) is a large-scale, multi-round survey conducted in a representative sample of households throughout India. Data on fertility, infant and child mortality, family planning use, maternal and child health, reproductive health, nutritional status, and use and quality of health and family planning services are collected at the national and state levels [26, 27].

To estimate the wealth- and education-related inequalities, we have used the data on wealth quintiles and the highest levels of education attained by women as provided by NFHS. The wealth quintiles are categorised into poorest, poorer, middle, richer and richest and are approximated based on assets and household characteristics. The education variable represents women’s highest level of education, grouped into the following four categories: no education, primary, secondary and higher education.

### Statistical analysis

Countdown to 2015 is a global movement initiated in 2005 that aims to monitor progress in reducing maternal and infant mortalities by introducing two summary measures: the Composite Coverage Index (CCI) and the co-coverage indicator [2, 28].

In this study, we examine inequalities in accessing RMNCH services using both indices. To ensure comparability of the indicators, the underlying indicators that make up these composite indices need to be the same ones. Due to constraints such as limited sample size and missing data in the NFHS dataset pertaining to care seeking for acute respiratory infections and oral rehydration salts for diarrhoea, which are both components in constructing the CCI, we opted to utilize the indicators that make up the co-coverage indicator. We modified the CCI by using the same indicators used to calculate the co-coverage indicator.

The indicators used in the construction of both the indices are: four or more Antenatal Care Visits (ANC), Tetanus Toxoid dosage during pregnancy (TT), Skilled Birth Assistance during childbirth (SBA), bacilli Calmette-Guerin vaccination (BCG), three doses of diphtheria, pertussis and tetanus vaccination (DPT3), Measles vaccination, Vitamin A supplementation (Vit A) and access to improved drinking water sources (AIW).

The CCI provides a weighted average of coverage during four stages in the continuum of care based on eight preventive and curative interventions. For the construction of the modified CCI, the interventions are classified as those for which the child, the mother and the household are the recipients [29]. The formula for the construction of the modified CCI is:

$$\frac13\;\ast\left(\frac{2\;\ast\;DPT3\;+\;MSL\;+\;BCG}4+\frac{Vit\;A\;+\;TT\;+\;\;ANC}3+\frac{SBA\;+\;AIW}2\right)$$

The co-coverage indicator captures the proportion of the population receiving most or all of the same eight selected RMNCH interventions [30, 31]. 

We were thus able to observe both the percentage of the ST population for whom a threshold (at least six indicators) of coverage is reached and the weighted average coverage for the ST population for the same eight indicators.

In the first part of the study, we determine the co-coverage indicator and the modified CCI for the ST population in Odisha and Jharkhand for NFHS-4 (2015-16) and NFHS-5 (2019-21). Subsequently, we examined absolute and relative wealth-and education-related inequalities among the ST groups in the two states for NFHS-4 (2015-16) and NFHS-5 (2019-21).

The magnitude of inequality is determined using the slope index of inequality (SII) and relative index of inequality (RII). SII measures absolute inequality through logistic regression, quantifying the difference in healthcare access between the highest and lowest wealth and education groups. Its values range from − 100 to + 100% points, with negative values indicating greater utilization/access among lower socioeconomic groups and positive values indicating the opposite.

The RII is defined as the ratio of the health outcome or indicator in the group at the highest wealth and education groups to the health outcome or indicator in the group at the wealth and education groups, while accounting for the entire socioeconomic distribution [32].

A generally accepted measure of analysing income inequalities in health is estimating differences across quintiles by a 20:20 ratio, where the health of the top 20% and the bottom 20% of the population in the income distribution are compared. However, this metric fails to capture the patterns of health status of the middle 60% of the population. The SII and RII together show the inequalities of the entire range of the population, thereby overcoming the limitations of other measures, including range, by reflecting the experiences of the entire population and are sensitive to the distribution of the population across socioeconomic groups [33].

The SII Is derived from the following simple linear regression model:

$$\mu=\beta_0+\beta_1R_jf\;orj=1-J$$

Where ${R}_{j}$ – Relative rank in the socioeconomic group (SEG) distribution

${\beta }_{0}$ – The estimated health status of a hypothetical person at the bottom of the hierarchy (${R}_{j}$ = 0)

Assuming linearity, SII is the difference between the average health status of the person at the bottom of the SEG (wealth and education) hierarchy and the person at the top. As SII is calculated for grouped data, weighted least squares are employed, with equal weights assigned to the population of group $j.$ The formula for SII [34] is

$$SII=\beta_1=\frac{\sum_{j\;=1}^J\;p_jR_j\;\left(\;\mu_{i-}\mu\right)}{\sum_{j\;=1}^J\;p_j\;R_j^2\;-\left(\sum_{j\;=1}^J\;p_jR_j\right)^2}$$

Where, ${\mu }_{j}$ – Average health status of SEG $j$

${p}_{j}$ – Population share of SEG $j$

${R}_{j}={\sum }_{\gamma }^{j-1}{p}_{\gamma -0.5{p}_{j}}$ – Relative rank of SEG $j$

$\mu ={\sum }_{j=1}^{J}{p}_{j}{\mu }_{j}$ – Average health status of the population

## Results

**Fig. 1:**
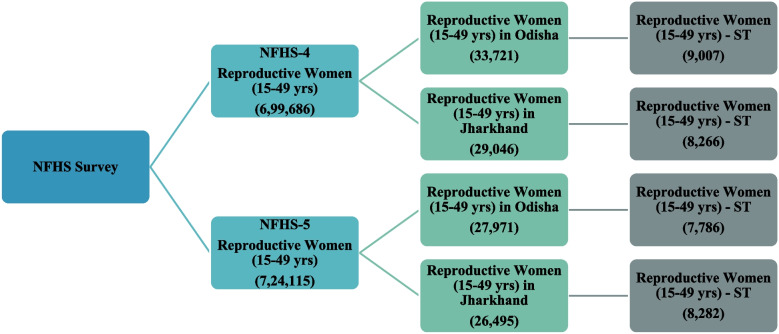
Sample size of ST population in Odisha and Jharkhand as to NFHS-4(2015-16) and NFHS-5 (2019-21). Source Authors’ estimate from NFHS-4 (2015-16) and NFHS-5 (2019-21)

Figure 1 shows the sample sizes used from NFHS-4 and NFHS-5 for this analysis.

### Section 3.1

**Fig. 2:**
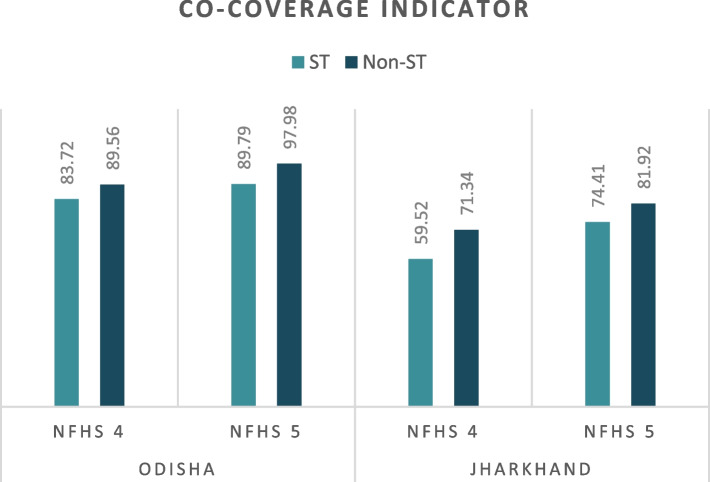
Comparison of co-coverage indicator of ST and non-ST women in Odisha and Jharkhand using NFHS-4 (2015-15) and NFHS-5 (2019-21). *Source* Authors’ estimate using fourth and fifth rounds of NFHS data

Figure 2 shows the comparison of the co-coverage indicator between ST and non-ST women in the two states between the fourth and fifth rounds of NFHS. The graph reveals that there has been a marked rise in co-coverage indicator two states among both groups. But the co-coverage is higher among non-ST women in the states of Odisha and Jharkhand, in both rounds of NFHS. Among ST women and children, the indicator has increased by 6 percentage points (p.p.), and 15 p.p. between NFHS-4 (2015-16) and NFHS-5 (2019-21) in Odisha and Jharkhand, respectively. The co-coverage indicator for non-ST women increased by 8 p.p., and 11 p.p. between the two rounds of NFHS in the two states.

**Fig. 3:**
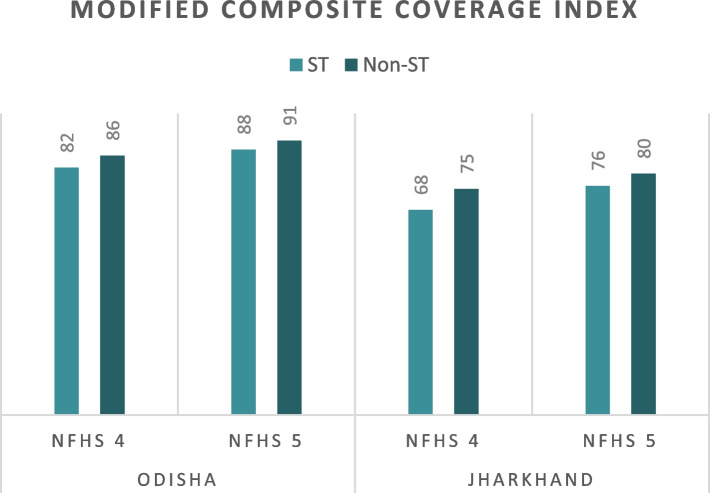
Comparison of modified CCI of ST and non-ST women in Odisha and Jharkhand using NFHS-4 (2015-15) and NFHS-5 (2019-21). *Source* Authors’ estimate using fourth and fifth rounds of NFHS data

Figure 3 compares the modified CCI of ST and non-ST populations in Odisha and Jharkhand between NFHS-4 (2015-16) and NFHS-5 (2019-21). Similar to the co-coverage indicator, the modified CCI indicates a higher average utilisation of RMNCH services by non-ST women compared to their ST counterparts. The increase in the modified CCI among the ST population is observed to be 6 p.p. and 8 p.p. in Odisha and Jharkhand, respectively, between NFHS-4 (2015-16) and NFHS-5 (2019-21). Among the non-ST counterparts, the increase is 5 p.p. for both states between 2016 and 2021.

Both the co-coverage indicator and the modified CCI are lower for Jharkhand among both ST and non-ST compared to Odisha. As measured by the co-coverage indicator, the performance of the two states in utilization of RMNCH services, both for ST and non-ST populations, is higher than that measured by the modified CCI (Figs. 2 and 3).

### Section 3.2

Tables 1 and 2 present the co-coverage and modified CCI for ST women belonging to the categorized wealth and education groups in Odisha and Jharkhand in NFHS-4 (2015-16) and NFHS-5 (2019-21). The study results indicate that women belonging to richer households and who are better educated, in general, have higher access to RMNCH interventions.

**Table 1:** Categorised co-coverage indicator and modified CCI for wealth quintiles in Odisha and Jharkhand

State	Wealth Quintile	Co-coverage indicator		Modified CCI	
		NFHS-4	NFHS-5	NFHS-4	NFHS-5
**Odisha**	Poorest	81.4	88.1	79.4	85.3
	Poorer	88.2	92.8	85.5	90.8
	Middle	93.3	93.8	88.8	92.0
	Richer	94.1	100.0	87.8	90.8
	Richest	90.0	100.0	93.5	97.2
**Jharkhand**	Poorest	54.5	73.6	64.4	74.2
	Poorer	72.6	76.1	75.6	80.9
	Middle	76.5	73.5	81.7	79.2
	Richer	72.7	90.1	85.7	87.6
	Richest	100.0	72.5	90.4	84.2

Source Authors’ estimate using NFHS

**Table 2:** Categorised co-coverage indicator and modified CCI for education groups in Odisha and Jharkhand

State	Educational group	Co-coverage indicator		Modified CCI	
		NFHS-4	NFHS-5	NFHS-4	NFHS-5
**Odisha**	No education	78.8	84.2	77.8	83.6
	Primary	90.1	97.3	84.9	90.3
	Secondary	90.9	93.1	87.1	89.6
	Higher	71.4	100.0	89.9	90.6
**Jharkhand**	No education	49.7	64.9	62.6	71.5
	Primary	62.0	77.6	68.2	76.5
	Secondary	71.8	82.1	75.9	80.1

Source Authors’ estimate using NFHS

Between NFHS-4 (2015-16) and NFHS-5 (2019-21), among all the wealth quintiles (except for the richest wealth quintile in Jharkhand), the co-coverage and the modified CCI values show an increase in both Odisha and Jharkhand. Among the richest wealth quintile in Jharkhand, we can observe a reduction of 27.5 p.p. and 6 p.p. in co-coverage indicator and modified CCI, respectively (Table 1).

Now observing the education groups, we see a similar pattern of increasing coverage of RMNCH services between the two rounds of NFHS, with an increase in co-coverage indicator and the modified CCI values (Table 2).

Among both the wealth and education categories we see that the co-coverage values are higher than the modified CCI values.

### Section 3.3

We have employed two complex measures to determine wealth and education-related inequalities, namely, SII, which discerns the average difference in utilisation across wealth and education groups and RII scores indicating the average ratio of utilisation across the groups. Table 3 displays the magnitude of wealth-related absolute and relative inequalities of the co-coverage indicator and modified CCI for Odisha and Jharkhand, for the two rounds of NFHS. The coefficient values of SII in both rounds of NFHS among the wealth groups are positive, indicating a higher coverage among wealthy households. From the table, we can observe that the absolute and relative inequalities in Odisha for co-coverage indicator have increased by 2 p.p. and 4 p.p. between NFHS-4 (2015-16) and NFHS-5 (2019-21), indicating an increase in wealth-related inequalities between the groups. With respect to modified CCI, the SII for wealth-related inequalities indicates an increase of 10 p.p. between NFHS-4 (2015-16) and NFHS-5 (2019-21), while the RII, an indicator of relative inequality, shows a reduction of 6 p.p. The SII and RII for the co-coverage index for Jharkhand between NFHS-4 (2014-15) and NFHS-5 (2019-21) present that the wealth-related absolute and relative inequalities have reduced by 38 p.p. and 8 p.p., respectively. While observing the trends in wealth- and education-related inequalities in Jharkhand for modified CCI, we can see a drastic reduction between 2016 and 2021. The absolute and relative wealth related inequalities have reduced by 18 p.p. and 32 p.p. between 2016 and 2021. Jharkhand has experienced a notable decrease in both SII and RII, indicating a more equitable distribution of wealth compared to Odisha (Table 3).

**Table 3:** Trends in wealth-related inequality in co-coverage indicator and modified CCI for Odisha and Jharkhand between NFHS-4 (2015-16) and NFHS-5 (2019-21)

Wealth-related inequality	Co-Coverage Indicator		Modified CCI	
**Odisha**	**SII**	**RII**	**SII**	**RII**
NFHS − 4	0.12* (-0.008,0.23)	1.13*** (0.98, 1.30)	0.02*** (0.11, 0.21)	1.20*** (1.13,1.28)
NFHS − 5	0.14*** (0.11,0.18)	1.17*** (1.12, 1.21)	0.12*** (0.06, 0.18)	1.14*** (1.07,1.22)
Change in inequality between NFHS-4 and NFHS-5	0.02	0.04	0.1	-0.06
**Jharkhand**	**SII**	**RII**	**SII**	**RII**
NFHS − 4	0.43*** (0.20, 0.67)	1.88*** (1.18, 2.59)	0.31*** (0.26,0.35)	1.50*** (1.40,1.61)
NFHS − 5	0.05 (-0.14, 0.27)	1.08*** (0.80, 1.36)	0.13*** (0.05,0.21)	1.18*** (1.06,1.29)
Change in inequality between NFHS-4 and NFHS-5	-0.38	-0.8	-0.18	-0.32

Source Authors’ estimates based on unit-level data of NFHS-4 (2015-16) and NFHS-5 (2019-21)

*P* value: * *p* < 0·10, ** *p* < 0·05, *** *p* < 0·01

(-) negative sign signifies a reduction in SII and RII values between 2015-16 to 2019-21

In Odisha, in education-related SII for co-coverage indicator, the RMNCH utilization is seen to be higher among the uneducated in NFHS-4 (2015-16), with a negative sign for the co-efficient. But inequalities increased with a pro-higher-educated-group bias in the fifth round by 27 p.p. Similarly, the RII for the co-coverage indicator in Odisha increased by 39 p.p. between the fourth and fifth rounds of NFHS. With respect to modified CCI in Odisha, between the education categories, both absolute and relative inequalities present a reduction of 7 p.p. and 10 p.p. respectively between NFHS-4 (2015-16) and NFHS-5 (2019-21) for modified CCI in Odisha. This is in direct contrast to what is observed using the co-coverage indicator, where the gap between the uneducated and those with a higher education increased. In the state of Jharkhand, the SII and RII values of education groups for co-coverage indicators are observed to have reduced by 18 p.p. and 53 p.p. between the fourth and fifth rounds of NFHS. Similarly, analysing education-related inequalities between the groups in modified CCI, we observe that the SII and RII values have reduced by 16 p.p. and 23 p.p. between NFHS-4 (2015-16) and NFHS-5 (2019-21). Jharkhand has consistently shown greater progress in reducing education-related inequality across all indicators than Odisha. Both states experienced a decrease in Modified CCI, but Jharkhand’s decrease was more pronounced, indicating more effective measures to reduce education-related disparities.

Comparing the absolute and relative inequalities in both co-coverage and modified CCI for the two states, the wealth- and education- related inequalities among the ST population in Jharkhand were much higher in 2015-16 that those in Odisha. However, these inequalities have fallen drastically in Jharkhand in 2019-21, bringing the inequality values close to those in Odisha (Table 4).

**Table 4:** Trends in education-related inequality in co-coverage indicator and modified CCI for Odisha and Jharkhand between NFHS-4 (2015-16) and NFHS-5 (2019-21)

Education-related inequality	Co-Coverage Indicator		Modified CCI	
**Odisha**	**SII**	**RII**	**SII**	**RII**
NFHS − 4	-0.08 (-0.40, 0.23)	0.90*** (0.56, 1.25)	0.15*** (0.12,0.18)	1.20*** (1.15,1.24)
NFHS − 5	0.19** (0.030, 0.35)	1.29*** (1.00, 1.56)	0.08*** (0.03,0.13)	1.10*** (1.03,1.16)
Change in inequality between NFHS-4 and NFHS-5	0.27	0.39	-0.07	-0.1
**Jharkhand**	**SII**	**RII**	**SII**	**RII**
NFHS − 4	0.37*** (0.34, 0.40)	1.81*** (1.70, 1.92)	0.28*** (0.22,0.33)	1.49*** (1.36,1.61)
NFHS − 5	0.19*** (0.02, 0.45)	1.28*** (1.00, 1.56)	0.12*** (0.09,0.14)	1.17*** (1.13,1.21)
Change in inequality between NFHS-4 and NFHS-5	-0.18	-0.53	-0.16	-0.23

Source Authors’ estimates based on unit-level data of NFHS-4 (2015-16) and NFHS-5 (2019-21)

*P* value * *p* < 0·10, ** *p* < 0·05, *** *p* < 0·01

‘(-) negative sign signifies a reduction in SII and RII values between 2015-16 to 2019-21

### Section 3.4

**Fig. 4:**
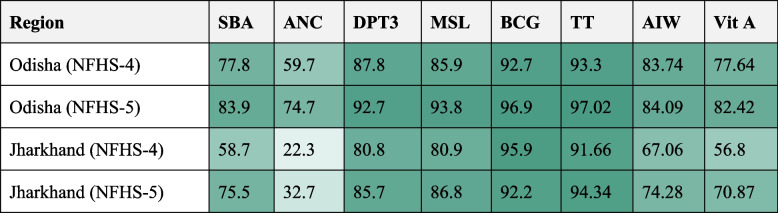
Heatmap for co-coverage and modified CCI indicators for Odisha and Jharkhand for NFHS-4 (2015-16) and NFHS-5 (2019-21). Source Authors’ estimate using NFHS

Figure 4 shows the heatmap for indicators of both indices for Odisha and Jharkhand for NFHS-4 (2015-16) and NFHS-5 (2019-21). The coverage for BCG, MSL, TT and DPT3 is shown to be high in the two states, with Odisha having more coverage when compared with Jharkhand. In Jharkhand, although the values exhibit an increase in the utilisation of four or more ANC between the fourth and fifth rounds, it is still very low, at 32.7% in NFHS-5.

**Fig. 5:**
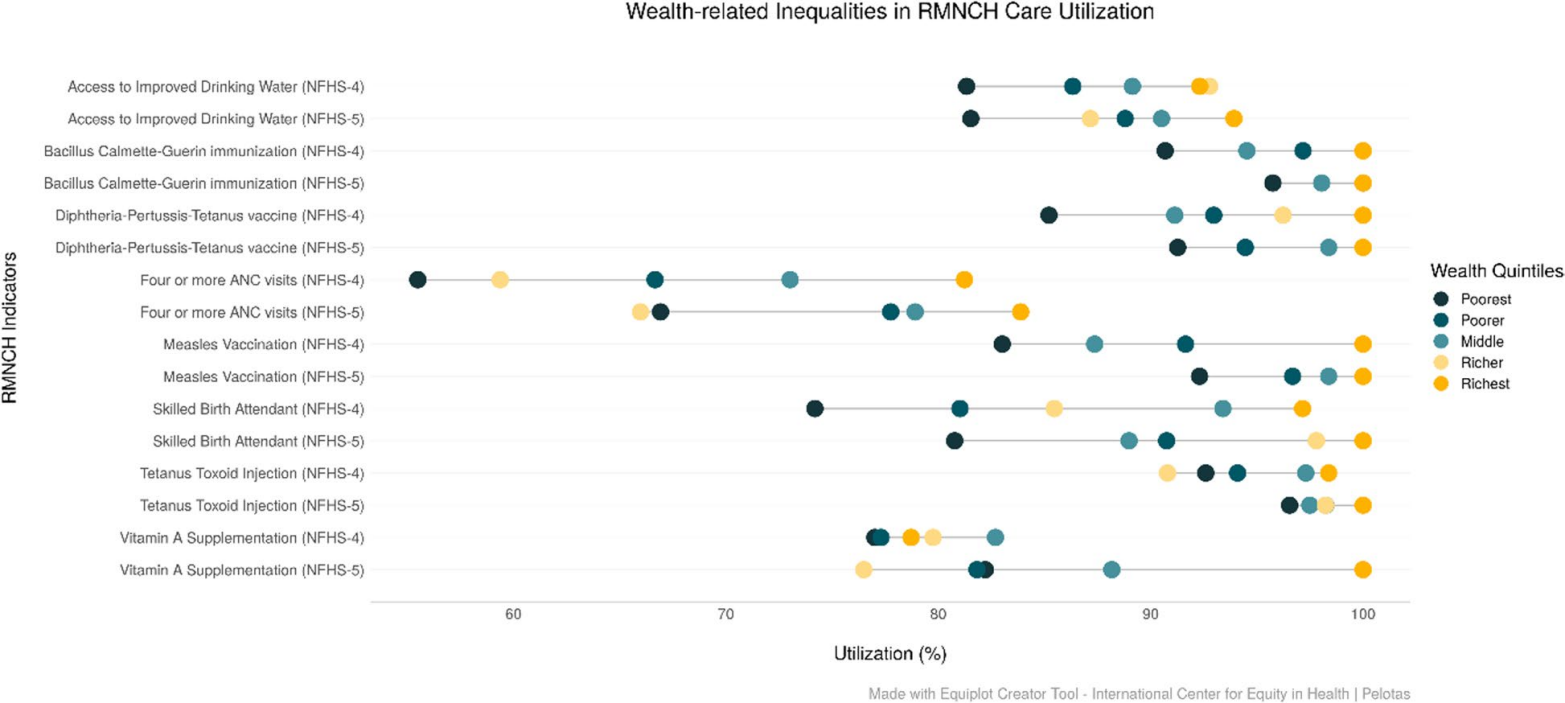
Trends in the coverage of RMNCH interventions among ST population across wealth quintiles in Odisha (NFHS-4 (2015-16) and NFHS-5 (2019-21)

We have employed equiplots to further analyse the utilization of RMNCH services by the components of the co-coverage indicator and the modified CCI for the ST population across wealth and education groups. They show the coverage level and gaps between the groups in NFHS-4 (2015-16) and NFHS-5 (2019-21) for each indicator used in the co-coverage indicator and the modified CCI for wealth quintiles and education categories. Overall, the utilization of RMNCH interventions is observed to be higher among ST women belonging to the richest wealth quintiles in Odisha. The inequalities between the highest and lowest wealth quintiles narrowed for BCG, DPT3, MSL and TT between NFHS-4 (2015-16) and NFHS-5 (2019-21). The magnitude of inequalities between rich and poor in IDW and Vitamin A supplementation exhibits an increase between 2016 and 2021. In NFHS-5 (2019-21), in four or more ANC services and Vitamin A supplementation, the richer quintile (fourth quintile) had the lowest utilization compared to other wealth quintiles (Fig. 5).

**Fig. 6:**
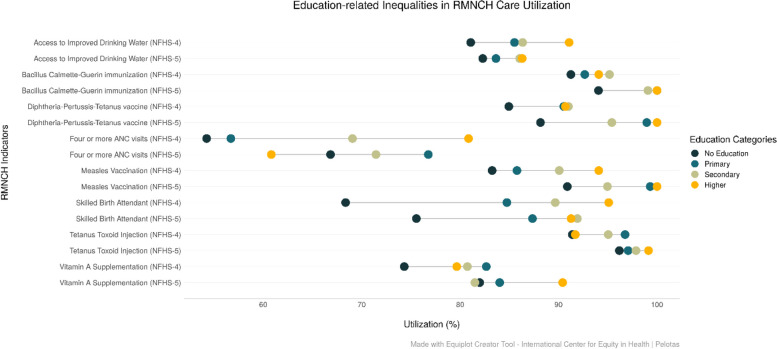
Trends in the coverage of RMNCH interventions among ST population across Education Groups in Odisha (NFHS-4 (2015-16) and NFHS-5 (2019-21)

The equiplots presenting education-related inequality show that the magnitude of inequality has decreased between the least and most educated women for IDW and SBA between NFHS-4 (2015-16) and NFHS-5 (2019-21) in Odisha. The inequality has broadened between the two rounds with respect to DPT3. In NFHS-4 (2015-16), TT and Vitamin A coverage was low for women with higher education compared to the lower-educated group. However, in NFHS-5 (2019-21), this pattern changed with women belonging to richest quintile and highly educated utilising most of these two interventions. We also observed that the highest educated had the least coverage for four or more ANC visits in NFHS-5 (2019-20), unlike NFHS-4 (2015-16) (Fig. 6).

**Fig. 7:**
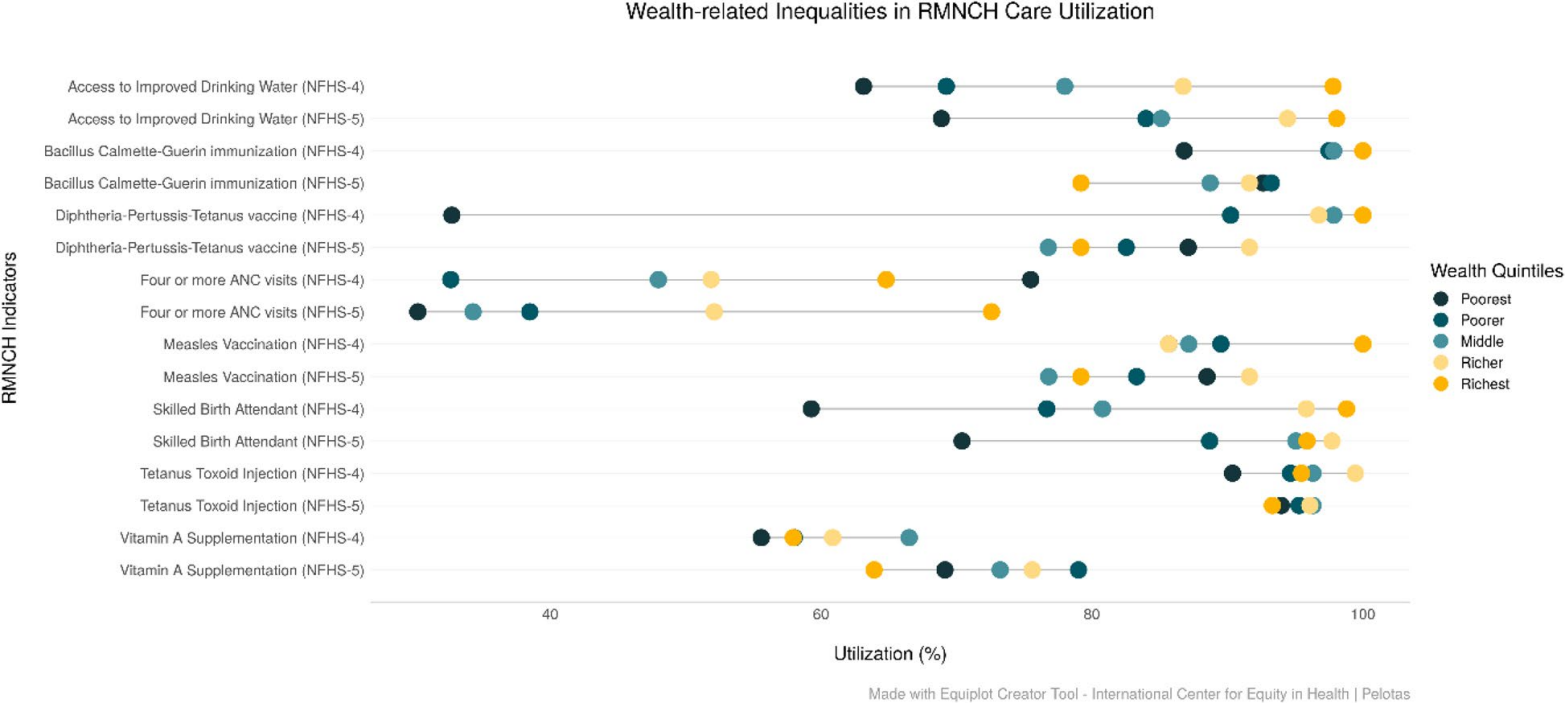
Trends in the coverage of RMNCH interventions among ST population across Wealth Quintiles in Jharkhand (NFHS-4 (2015-16) and NFHS-5 (2019-21)

**Fig. 8:**
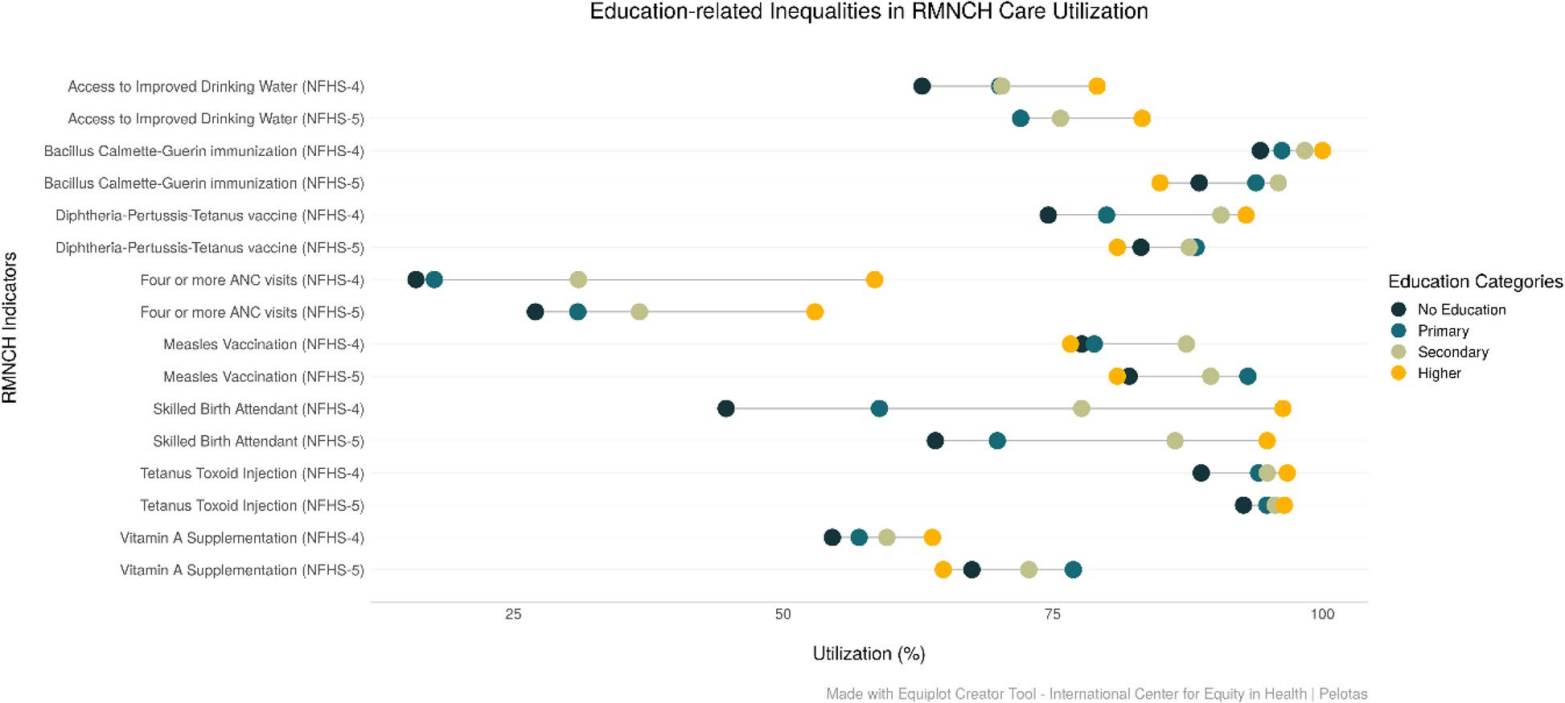
Trends in the coverage of RMNCH interventions among ST population across Education Groups in Jharkhand (NFHS-4 (2015-16) and NFHS-5 (2019-21)

Figures 7 and 8 present the equiplots of RMNCH utilization by each component of the co-coverage indicator and the CCI by wealth quintile and education group in Jharkhand. The inequality between the rich and poor shows a reduction between NFHS-4 (2015-16) and NFHS-5 (2019-21) for IDW and SBA. We also note that, as to NFHS-5 (2019-21), the utilization of BCG, TT and Vitamin A supplementation, is lowest among the richest (fifth) wealth quintile. The utilisation of DPT3 and MSL is also observed to be low among the richest wealth groups in Jharkhand in 2019-21. 4 or more ANC visits was highest among the poorest in NFHS-4 but this was reversed in NFHS-5 (Fig. 7).

With respect to education-related inequalities, the magnitude of inequalities has narrowed for four or more ANC visits, SBA and TT between the most and the least educated ST women. In the figure, we observe that the utilization of BCG, DPT3, MSL and Vitamin A supplementation is lowest among the higher education groups compared to other education categories in NFHS-5 (Fig. 8).

## Discussion

Maternal mortality and underutilisation of RMNCH services remain significant challenges among ST communities in India. ST women often face multiple barriers that hinder their access to quality health services, leading to higher pregnancy-related mortality and morbidity in the community. Limited availability of healthcare facilities, inadequate infrastructure, geographical remoteness, cultural and language barriers, and socio-economic disparities contribute to this issue [35]. Additionally, traditional beliefs and practices related to childbirth and limited awareness about modern healthcare further exacerbate the problem [36]. A lower literacy level is also a leading factor contributing to reduced healthcare services utilization by ST women. The literacy level among the ST population in Odisha and Jharkhand is observed to be 52.2 per cent and 57.1 per cent, respectively [37]. This percentage is well below the average literacy level of India, which stands at 74.04 per cent [14].

The existing body of scholarship has sought to capture the disparities in healthcare service utilization and health outcomes between tribal and non-tribal communities in India. These studies consistently reveal that non-tribal populations utilize more health services and enjoy favourable health outcomes in India compared to their tribal counterparts [38–42]. Despite the differences existing between tribal and non-tribal populations, there exist disparities within the tribal communities as well. In recent tribal health research, efforts have been made to delve into and capture specific intra-group differences in health outcomes and health services utilization within tribals across different states in India [43–46].

However, in the general understanding, ST communities are considered a homogenous group, and the differences existing within them are not considered when designing policies. Our study attempts to address this oversight by examining how wealth and education impact the utilisation of RMNCH services within ST communities in the states of Odisha and Jharkhand in India. We capture the magnitude of these differences by employing two rounds of NFHS data.

Summary measures such as the co-coverage indicator and the CCI combine information from various RMNCH indicators along the continuum of care, providing a comprehensive assessment of healthcare access and utilisation [30]. The objective of summary measures is to provide a more precise statistical representation of RMNCH coverage compared to standalone indicators, with the aim of easier understanding of overall trends and patterns, including disparities and inequalities in healthcare provision. There exists a high correlation between these two measures because of the common interventions used in determining both the co-coverage indicator and the modified CCI. However, the interpretation and primary uses of each differ. The co-coverage indicator is a cumulative measure, which is estimated at the individual mother and child level, whereas the derivation of modified CCI is at a group level, calculated as the average of the eight interventions [28].

The results of our study corroborate the existing literature on wealth-related inequalities in RMNCH coverage, which indicates a higher utilisation of services by women belonging to the highest wealth quintile. Sumirtha et al. conducted a similar analysis employing the CCI and Co-coverage indicator, revealing varying levels of inequality in RMNCH service utilization across and within different states in India. In the study, among the total population in Odisha (inclusive of all social groups), the absolute and relative inequalities in the utilization of RMNCH services for CCI and co-coverage indicator stood at 0.11 and 1.16 and 0.27 and 1.50, respectively [6]. Leventhal et al., in a broader assessment of 36 countries, observed a pro-rich distribution pattern in RMNCH coverage across most interventions, with higher coverage among wealthier segments, except for two breastfeeding indicators favouring poorer women and children [9]. Shirisha et al. explored wealth-related disparities in RMNCH intervention coverage in Tamil Nadu and Chhattisgarh, highlighting greater service utilization among individuals from wealthier households, albeit with a decreasing trend in inequalities over a decade [47].

The study results show that, in Odisha and Jharkhand, the women belonging to non-ST communities utilised RMNCH services more than their ST counterparts. The co-coverage indicator and modified CCI values are higher among ST women in Odisha than in Jharkhand. In NFHS-5 (2019-21), a difference of 8.2 p.p. and 7.5 p.p. is observed between the ST and non-ST groups with respect to the co-coverage indicator in Odisha and Jharkhand, respectively. In modified CCI, between the two groups in Odisha and Jharkhand, a difference of 3 p.p. and 4 p.p. exists in 2019-21. However, we observe an increased co-coverage and modified CCI in both the groups in 2019-21 compared to 2015-16, indicating a higher utilization of RMNCH services over the years.

In the analysis of wealth- and education-related inequalities in RMNCH services utilization within ST women, the findings revealed the existence of significant disparities based on wealth and education, with women from wealthy families and those with higher levels of education having better access to the services in general. Firstly, the observed results of the co-coverage indicator and modified CCI between the five wealth quintiles show higher index values among the women belonging to higher wealth quintiles than lower wealth quintiles in Odisha and Jharkhand. Similarly, among the education categories, we observe a pattern with highly educated women having higher index values, indicating a greater utilization of RMNCH services by more educated ST women than the least educated ones. However, we observe an exception, where in Jharkhand, the co-coverage indicator value was lowest for the richest quintile in NFHS-5 (2019-21) compared to other groups.

The results of SII and RII exhibit that in Odisha, absolute and relative inequalities by wealth, according to both the co-coverage indicator and the modified CCI, increased between the fourth and fifth rounds of NFHS. The SII value shows an increase of 2 p.p. and 1 p.p. for co-coverage and modified CCI values between 2016 and 2021. However, it is noteworthy that despite these widening disparities, there has been a narrowing of inequality between rich and poor groups concerning certain interventions such as BCG, DPT3, MSL, and TT. Conversely, the utilization rates of interventions like IDW and Vitamin A supplementation have exhibited considerable expansion between NFHS-4 (2015-16) and NFHS-5 (2019-21), surpassing the reduced disparities observed for the aforementioned four interventions. This disparity in utilization rates has consequently led to an overall escalation in the magnitude of the two indices utilized. The observed reductions in the utilization of specific interventions can be the result of COVID-19 pandemic and the associated lockdowns. One study found a significant reduction in paediatric and neonatal care utilisation during the pandemic in a tertiary care hospital in Odisha [48]. We infer that these impacts must have been felt even more severely among the poorest of the tribal population.

Similarly, we notice a marked rise in education-related inequalities between the two rounds according to the co-coverage indicator and a decrease according to the modified CCI in Odisha. This could indicate that the group-level coverage became more equitable, while individual mother-child pairs at the lowest educated levels fared worse in 2019-21. The increased inequality can result from the changed pattern in the utilization of TT and Vitamin A supplementation between NFHS-4 (2015-16) and NFHS-5 (2019-21). The utilization of these two interventions was higher among lower wealth quintiles in 2015-16 than higher wealth quintiles, whereas in 2019-21, this changed with rich and highly educated women utilising these services far more than their counterparts. Furthermore, there has been an expansion noted in the utilization of DPT3 between rich and poor between the two rounds.

However, in the state of Jharkhand, a different pattern is observed, with both the absolute and relative inequalities falling drastically with respect to both wealth and education between NFHS-4 (2015-16) and NFHS-5 (2019-21). Even though inequalities were high in Jharkhand compared to Odisha in NFHS-4 (2015-16), these reduced drastically in NFHS-5 (2019-21), bringing Jharkhand on par with Odisha. We observe two reasons for the reduction in the inequalities. In NFHS-5 (2019-21), the utilization of BCG, TT, Vitamin A supplementation DPT3 and MSL among the rich wealth group has fallen behind the poor wealth quintiles. Similarly, a changed pattern in the utilization of BCG, DPT3, MSL and Vitamin A supplementation is observed in NFHS-5 (2019-21) where the least educated utilised more of these services. These changed patterns in the distribution of the utilization among the wealth quintiles and education groups in NFHS-5 (2019-21) compared to NFHS-4 (2015-16) has contributed significantly to the reduction in the inequalities in Jharkhand.

In 2005, the Government of India implemented the Janani Suraksha Yojana (JSY), a conditional cash transfer program aimed at encouraging women to deliver their babies in healthcare facilities. Over time, JSY has succeeded in increasing institutional deliveries, particularly among the most marginalized communities in underperforming states in India [49, 50]. However, various studies have pointed out a pattern of inequity and disparity in JSY’s accessibility and coverage among women from disadvantaged backgrounds, primarily due to supply-side obstacles [50–54]. Furthermore, research indicates a significant discrepancy in the utilization of JSY between states, with Jharkhand exhibiting a utilization rate of 41% compared to Odisha’s 73% [27]. A study examining the utilization of JSY across different socio-economic groups in Odisha and Jharkhand found that in both states, wealthier women tend to utilize the program more than their economically disadvantaged counterparts [49].

In Jharkhand, only 75.5% of Scheduled Tribe (ST) women have delivered with skilled birth assistance, compared to 83.9% in Odisha. The introduction of a conditional cash transfer programme, MAMATA scheme by the state government of Odisha has significantly contributed to the increased uptake of maternal health care services, in specific, institutional deliveries in Odisha [40, 55]. A study by Saha and Paul (2021), observed that the major factors which may have constrained women from Jharkhand utilising maternal health care services are low exposure to mass media, distance to the health facilities, lack of awareness of the importance of institutional deliveries, lack of transportation and increased health care costs [56]. This variance can also be attributed to the disparity in healthcare infrastructure between Odisha and Jharkhand. According to the National Health Profile 2022, Odisha has a higher number of Primary Health Centres (PHCs) and Community Health Centres (CHCs) compared to Jharkhand. Specifically, Odisha has 1379 PHCs and 384 CHCs, whereas Jharkhand has 350 PHCs and 176 CHCs [57].

Of greatest concern was the proportion of tribal women in Jharkhand who visited an ANC at least 4 times. This was at 22.2% in NFHS-4 and at 32.7% in NFHS-5. Ensuring at least four visits to an Antenatal Care Centre (ANC) has been shown to be a major intervention, helping avoid maternal and neonatal deaths and is recommended by WHO’s Global Strategy for Women’s, Children’s and Adolescents’ Health [58]. During these visits, women are educated about possible pregnancy and delivery complications; provided with iron and folic acid supplements that prevent anaemia; received help for hypertension, which can prevent eclampsia; and immunised against tetanus and other endemic diseases [59].

Among the RMNCH indicators, vaccination coverage has shown remarkable success rates. BCG vaccination coverage stands above 92%, TT above 91%, and DPT3 and MSL above 80%. This success can largely be attributed to the Universal Immunization Program (UIP) initiated by the Immunization Division under the National Health Mission in 1985. The UIP offers free immunization against 12 vaccine-preventable diseases [60]. In 2014, Mission Indradanush was launched with the ambitious goal of achieving 90% full immunization coverage, particularly targeting hard-to-reach areas. This mission was rolled out in 12 states, including Odisha and Jharkhand, in its initial phase. Additionally, an electronic vaccine intelligence network was implemented to enhance the monitoring of vaccine stocks and logistics [61]. A significant contributor to the increased vaccination coverage has been the role of Accredited Social Health Activists (ASHAs), who are tasked with ensuring mothers adhere to the vaccination schedule for their children. Studies have highlighted ASHAs’ pivotal role in this regard [61]. Furthermore, the introduction of Janani Suraksha Yojana (JSY) has been associated with increased vaccination rates for BCG and DPT, which are typically administered around birth. However, the impact of JSY seems to diminish for measles vaccination, which is administered a few months after birth [62].

The study findings underscore the urgent need for targeted interventions and policies to address the inequalities in accessing RMNCH services among ST communities in Odisha and Jharkhand. This paper makes two significant contributions: First, it establishes that the poor and the uneducated among the ST population in the two states suffer to a greater extent from lack of access to RNMCH services. The empirical part of this paper provides the extent to which relative and absolute inequities exist in these populations. The second important contribution of this paper is to suggest an analysis of the utilisation of RMNCH services from the primary records of household members maintained at primary health care facilities and complement it with qualitative research on households’ behaviour to assess their utilization levels, according to their social and economic factors. Policymakers will thus have a better insight on how to further refine and redesign their awareness campaign and create higher demand for these services from these sub-populations.

## Conclusion

This study evaluates the wealth- and education-related inequalities in access to RMNCH services within ST communities in Odisha and Jharkhand. The study results indicate a higher access to reproductive and maternal healthcare services for women who are better educated and belong to richer households. In Odisha, inequalities between rich and poor households widened for both modified CCI and co-coverage indicator over the five years between 2016 and 2021, while the gap narrowed over the same period in Jharkhand. In Odisha, the inequalities in utilisation of interventions, including IDW and Vitamin A supplementation indicate a widening between NFHS-4 (2015-16) and NFHS-5 (2019-21), which would have possibly contributed to the increased magnitude of absolute and relative inequalities in RMNCH utilization over the years. However, a small reduction in the inequalities between the rich and poor is observed for interventions including BCG, DPT3, MSL and TT. In Jharkhand in NFHS-5 (2019-21), a changed pattern is observed in the utilization of certain interventions, including BCG, TT, Vitamin A Supplementation, DPT3, and MSL, where the poorest quintiles utilised more of these services than their richer counterparts. This is observed to be the major reason for the reduced wealth-related inequalities in the state. With respect to education-related inequality, both absolute and relative inequalities in modified CCI decreased for both states. However, with respect to co-coverage indicator, inequalities exhibited an increase in Odisha and a reduction in Jharkhand. There are two noticeable reasons for the increase in inequality in Odisha according to co-coverage indicator. First, an observable widening in the gap between the rich and poor with respect to utilization of DPT3 is seen between 2015-16 and 2019-21. Secondly, a changed pattern in the utilization of TT and Vitamin A supplementation is observed in NFHS-5 (2019-21), where the least educated utilised more of these services compared to the highly educated women. This is different from NFHS-4 (2015-16), where highly educated women utilised more of these services in Odisha. The data indicates that in Odisha, ST women exhibit higher coverage of RMNCH services compared to those in Jharkhand. There is a notable need for improved coverage of Antenatal Care (ANC) services in Jharkhand, with estimates suggesting a significantly low utilization rate, with only one-third of the total ST population accessing these services. However, immunization rates, including for BCG, MSL, TT, and DPT3, performed well in both states, showing improvement between 2015-16 and 2019-21. Therefore, addressing the socio-economic determinants of health, such as poverty, education, and social exclusion, is vital for achieving equitable healthcare access for ST communities.

Policies should uplift marginalised populations by providing targeted social support, educational opportunities, and economic empowerment initiatives. Along with this, it is important to conduct regular evaluations and make necessary adjustments based on the feedback and experiences of the community to ensure the effectiveness and sustainability of these policies.

The limitations of this study are related to those that arise because of the nature of the NFHS data sets and of the indicators we have used. The NFHS collects cross-sectional data, and so trends are to be approached with caution. When sub-categorising the ST population by wealth and education, each sub-group only has a small number of respondents. We have accounted for this by using sampling weights in all our calculations. In the construction of the modified CCI, we follow the literature and assign equal weightage to each of the eight interventions. Conceptually, weighting each component commensurate to its impact on health status would be more reflective of the true value of coverage, and would have yielded better insights into inequality. In the absence of this data, we approximate the value by assigning equal weights to each indicator.

## Data Availability

The datasets generated during and/or analysed during the current study are available in the DHS and IIPS website, accessed from https://dhsprogram.com/data/.
